# PP63 Predictive Value Of Intermediate Endpoints For Overall Survival In High-Risk Localized And Locally Advanced Prostate Cancer

**DOI:** 10.1017/S0266462325102821

**Published:** 2025-12-29

**Authors:** Uwe Siebert, Francesco De Solda, Kimberly Hofer, James McCallion, Sharon A. McCarthy, Suneel D. Mundle, Mir-Masoud Pourrahmat, Victoria Wan, Beate Jahn

## Abstract

**Introduction:**

Demonstrating meaningful overall survival (OS) treatment benefit in high-risk localized or locally advanced prostate cancer (HR-LPC/LAPC) remains challenging due to the prolonged natural disease evolution. There is growing interest in validating intermediate clinical endpoints (ICEs) for OS. We evaluated the predictive value of ICEs for OS in HR-LPC/LAPC using data from randomized controlled trials (RCTs) and non-randomized comparative studies (non-RCTs).

**Methods:**

We conducted a systematic literature review using Embase, MEDLINE, CENTRAL, and gray literature (22 March 2024) to identify RCTs and non-RCTs investigating therapeutic options for adults (≥18 years) with HR-LPC/LAPC. Eligible studies reporting treatment effects (hazard ratios [HR]) for OS (HR_OS_) or ICEs were included. Event-free survival (EFS), metastasis-free survival (MFS), no evidence of disease (NED), and pathological complete response (pCR) were ICEs of interest. Correlation of each ICE with OS was evaluated using Bayesian bivariate random-effects meta-analysis (BRMA) with an uninformative prior distribution. The predictive value of each ICE was determined by assessing observed versus predicted treatment effects of OS via leave-one-out cross validation (LOOCV).

**Results:**

We selected 137 unique studies (89 RCTs and 48 non-RCTs) for meta-analysis: EFS-OS, k=85 studies (n=53,965 patients); MFS-OS, k=79 (n=51,746); NED-OS, k=100 (n=56,187); pCR-OS, k=112 (n=70,228). We observed moderate correlation estimates of 0.53 (95% credible interval [Crl]: −0.23, 0.96), 0.69 (95% CrI: −0.35, 0.98), and −0.53 (95% CrI: −0.95, 0.39) for EFS-OS, MFS-OS, and NED-OS, respectively. pCR-OS showed a weak correlation (0.06, 95%Crl: −0.89, 0.94). None of the correlation estimates were statistically significant. An RCT-only sensitivity analysis showed consistent results. The LOOCV had good predictive accuracy, with 95 percent prediction intervals capturing the observed HR_OS_ for 89.5, 87.5, 100, and 83.3 percent of studies, for respective ICEs. No ICEs met the Institute for Quality and Efficiency in Health Care (IQWiG) criteria for strong correlation.

**Conclusions:**

Despite consistent direction of associations between ICEs and OS using rigorous BRMA, we could not meet the stringent surrogacy thresholds used by health technology assessment (HTA) agencies. Our findings highlight a need for further analysis to prove correlations, including analyses using real-world data and consideration of how surrogacy evidence is assessed by HTA agencies in early-stage prostate cancer.

